# A Case of Recurrence of Fibrolamellar Hepatocellular Carcinoma After 18 Years Underscores the Need for Extended Patient Surveillance

**DOI:** 10.7759/cureus.64156

**Published:** 2024-07-09

**Authors:** Elsa Hallab, Mark Yarchoan, Marina Baretti

**Affiliations:** 1 Oncology, Sidney Kimmel Comprehensive Cancer Center, Johns Hopkins Medicine, Baltimore, USA; 2 Oncology, Gastrointestinal Cancer, Sidney Kimmel Comprehensive Cancer Center, Johns Hopkins Medicine, Baltimore, USA

**Keywords:** fibrolamellar, liver cancer, cancer surveillance, hepatocellular carcinoma (hcc), fibrolamellar hepatocellular carcinoma

## Abstract

Fibrolamellar hepatocellular carcinoma (FLC) is a rare form of primary liver cancer that predominantly affects adolescents and young adults with no history of cirrhosis. Surgical resection is a potentially curative treatment option, but the optimal duration of surveillance after a potentially curative surgical resection is not known. Here, we present a case of a patient with FLC who developed a recurrence of FLC nearly two decades after resection of the primary tumor. Although the optimal duration of imaging surveillance for FLC is not known, this case report provides initial evidence that long-term surveillance in patients with FLC who have received curative intent surgery is warranted.

## Introduction

Fibrolamellar hepatocellular carcinoma (FLC) is a rare form of primary liver cancer with distinct molecular, pathological, and clinical features from other forms of primary liver cancer. It accounts for less than 1% of all primary hepatic malignancies [1]. FLC predominantly affects adolescents and young adults with no history of liver disease or cirrhosis. The putative oncogenic driver is a recurrent in-frame fusion of exon 1 of DNAJB1 with exons 2-12 of PRKACA, yielding a chimeric-translated protein, DNAJ-PKAc.

For the subset of patients with FLC who are identified at an early stage, surgical resection may be an effective treatment strategy [2]. However, more than 50% of patients who undergo surgical resection for FLC eventually recur in historical series [3]. For patients with unresectable FLC, there is no approved or standardized systemic treatment. Patients with FLC have generally been excluded from registrational clinical trials of systemic therapy for HCC, as it is recognized that FLC is a distinct entity and generally demonstrates primary resistance to therapies developed for other primary hepatic malignancies.

Contemporary guidelines for hepatobiliary malignancies from the National Comprehensive Cancer Network (NCCN) and the American Association for the Study of Liver Diseases (AASLD) do not provide management recommendations for FLC [4,5]. However, the treatment of FLC is sometimes extrapolated from practice patterns developed for other primary malignancies in clinical practice. Contemporary treatment guidelines for biliary tract cancers (BTC) and hepatocellular carcinoma (HCC), the two most common forms of primary liver cancer, generally recommend at least five years of surveillance following curative intent resection. Whether five years is an appropriate endpoint for the surveillance of FLC is still not clear.

Here, we present a case of a patient with FLC whose cancer recurred 18 years after resection of the primary tumor. This case report highlights the risk for late recurrences and the need for prolonged FLC surveillance after potentially curative resection.

The patient provided written consent for the publication of this report (IRB Number IRB00377265).

## Case presentation

A 30-year-old, White male patient presented to the emergency room in late 2023 with sudden-onset abdominal pain. His past medical history was notable for a remote history of FLC, which was initially diagnosed when he was 12 years old. During his original FLC diagnosis, he presented to medical attention with abdominal pain and was found to have elevated liver function tests (LFTs). Magnetic resonance imaging (MRI) revealed a 7.0x6.6 cm hypointense mass with central scarring. The patient underwent central hepatic lobe resection in late 2005 and pathology was consistent with FLC (stage pT1bN0M0). He did not receive any adjuvant therapy. He was followed with surveillance imaging through 2016 without evidence of recurrence, after which monitoring was stopped.

**Figure 1:**
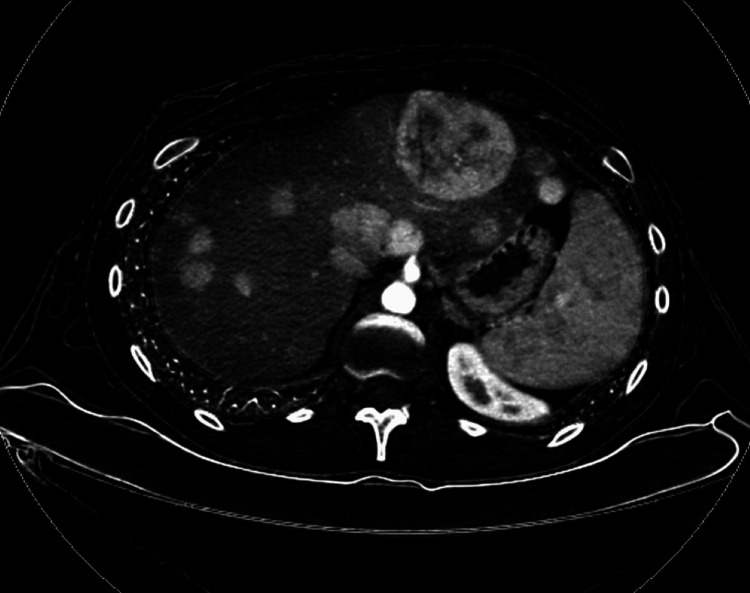
CT scan of the subject after a recurrence of FLC was recognized The patient had discontinued regular surveillance scans at year 10 after his initial resection and presented with a substantial and symptomatic disease burden at year 18 as illustrated in the scan shown. FLC: fibrolamellar carcinoma; CT: computerized tomography

In the emergency room, during the current evaluation of his abdominal pain in 2023, contrast-enhanced computed tomography (CECT) showed numerous hypodense and enhancing hepatic masses, as well as a soft tissue mass in the gastrohepatic ligament (Figure 1). MRI identified multiple solid enhancing masses with near uniform washout throughout the postoperative liver, which included a 3.8 cm mass in segment 2, a 2.0 cm mass in segment 1, and a 2.3 cm mass in segment 6. Laboratory studies showed preserved liver function (alanine aminotransferase (ALT), 56 U/L; aspartate aminotransferase (AST), 31 U/L; albumin, 4.8 g/dL; bilirubin total, 0.53 mg/dL; alkaline phosphatase (ALP), 71 U/L; prothrombin time (PT), 14.7 seconds; INR, 1.28). Tests for tumor markers revealed the following: cancer antigen (CA) 19-9, 20.55 U/mL; alpha-fetoprotein (AFP), <2.2 ng/mL; and carcinoembryonic antigen (CEA), 1.93 ng/mL, which were all within their respective normal ranges.

The lesion in the right liver was biopsied in 11/2023 and confirmed the diagnosis of FLC. A subsequent biopsy of this right hepatic lesion done in 04/2024 also confirmed FLC, and molecular testing (performed by BostonGene, Waltham, MA, US) confirmed the presence of an in-frame DNAJB1-PRKACA fusion.

## Discussion

FLC is a rare form of primary liver cancer, for which optimal management paradigms have not been established. Complete surgical resection is the only potentially curative option, but the optimal duration of surveillance after a potentially curative surgical resection is not known. In a prior retrospective study, patients with FLC undergoing curative-intent surgery had a median overall survival and recurrence-free survivals at 5 years of 62% and 45% [6]. In this prior retrospective study, in the subset of patients who remained without recurrence at Year 4, none subsequently recurred.

While there are no guidelines for FLC screening, and FLC is a distinct form of liver cancer, clinical practices may be influenced by guideline recommendations for other primary liver cancers. Contemporary guidelines for other primary liver cancer generally recommend at least five years of surveillance (Table 1), although HCC usually arises in the setting of liver cirrhosis, and patients with cirrhosis are recommended to continue indefinite screening to identify new liver cancers. FLC does not usually occur in the setting of cirrhosis, and therefore such recommendations would not apply. As FLC bridges pediatric and adult cancers, clinical practice may also be impacted by treatment paradigms for other pediatric solid cancers. Surveillance imaging in the pediatric population differs depending on the malignancy, but for several pediatric cancers imaging surveillance is not routinely recommended beyond Years 5-10.

**Table 1:** Imaging recommendations for hepatocellular and biliary tract cancer surveillance NCCN: National Comprehensive Cancer Network; AASLD: American Association for the Study of Liver Diseases

Journal	Type of cancer	Duration of surveillance imaging
NCCN	Hepatocellular Carcinoma	“At least 5 years; and thereafter screening is dependent on HCC risk factors” [4]
NCCN	Biliary Tract Cancer	“Up to 5 years, or as clinically indicated" [4]
AASLD	Hepatocellular Carcinoma	“The optimal timing and duration of surveillance after surgical resection is unknown, although AASLD recommends indefinite surveillance" [5]

Although the optimal duration of imaging surveillance is not known, this case report provides initial evidence that screening for FLC should continue longer than is currently recommended for other forms of primary liver cancer. Although advanced FLC can progress rapidly, it classically recurs soon after resection and has a median survival of only 12-14 months. This case report demonstrates the heterogeneity of FLC and the potential for FLC to progress slowly, resulting in late recurrences.

A limitation of the present case report is that we were unable to obtain tissue from the original FLC surgery and thus are unable to confirm the presence of shared mutations to confirm at a molecular level that the two FLCs are indeed the same cancer. Given the rarity of FLC (0.185/100,000 individuals within the United States [7]), it is statistically extremely unlikely that the same patient would spontaneously develop FLC twice in the same lifetime. While we presume that the same cancer recurred, we cannot exclude the possibility that this patient harbored a liver field defect (for example, the presence of a DNAJB1-PRKACA fusion in the background liver) that resulted in the development of two independent FLC tumors.

## Conclusions

In conclusion, this case of FLC recurrence almost two decades after the initial diagnosis underscores the imperative for long-term surveillance in patients with FLC who have received curative intent surgery. Oncologists and primary care physicians should be aware of this potential for late recurrence and should inform patients of the importance of ongoing surveillance. Future research is needed to establish specific long-term follow-up protocols and to identify potential markers that could predict recurrence risk, ultimately guiding tailored surveillance strategies.
